# Initial clinical experience with magnetic resonance-guided radiotherapy in pediatric patients: Lessons learned from a single institution with proton therapy

**DOI:** 10.3389/fonc.2022.1037674

**Published:** 2023-01-11

**Authors:** Matthew D. Hall, Kathryn E. Mittauer, Roberto Herrera, Katherine Von Werne, Rupesh Kotecha, Noah S. Kalman, James McCulloch, Diane Alvarez, Nicole C. McAllister, Delia G. Doty, Amy E. Rzepczynski, Will Deere, Alonso N. Gutierrez, Michael D. Chuong

**Affiliations:** ^1^ Department of Radiation Oncology, Miami Cancer Institute, Baptist Health South Florida, Miami, FL, United States; ^2^ Herbert Wertheim College of Medicine, Department of Radiation Oncology, Florida International University, Miami, FL, United States

**Keywords:** stereotactic body radiation therapy, SBRT, SABR, MR Linac, motion management, anesthesia, proton therapy, adaptive replanning

## Abstract

**Purpose/Objectives:**

Magnetic resonance-guided radiotherapy (MRgRT) is increasingly used in a variety of adult cancers. To date, published experience regarding the use of MRgRT in pediatric patients is limited to two case reports. We report on the use of MRgRT for pediatric patients at our institution during a four-year period and describe important considerations in the selection and application of this technology in children.

**Materials/Methods:**

All patients treated with MRgRT since inception at our institution between 4/2018 and 4/2022 were retrospectively reviewed. We also evaluated all pediatric patients treated at our institution during the same period who received either imaging or treatment using our magnetic resonance-guided linear accelerator (MR Linac). We summarize four clinical cases where MRgRT was selected for treatment in our clinic, including disease outcomes and toxicities and describe our experience using the MR Linac for imaging before and during treatment for image fusion and tumor assessments.

**Results:**

Between 4/2018 and 4/2022, 535 patients received MRgRT at our center, including 405 (75.7%) with stereotactic ablative radiotherapy (SABR). During this period, 347 distinct radiotherapy courses were delivered to pediatric patients, including 217 (62.5%) with proton therapy. Four pediatric patients received MRgRT. One received SABR for lung metastasis with daily adaptive replanning and a second was treated for liver metastasis using a non-adaptive workflow. Two patients received fractionated MRgRT for an ALK-rearranged non-small cell lung cancer and neuroblastoma. No Grade 2 or higher toxicities were observed or reported during MRgRT or subsequent follow-up. Twelve patients underwent MR imaging without contrast during treatment for brain tumors to assess for tumor/cystic changes. Two patients treated with other modalities underwent MR simulation for target volume delineation and organ at risk sparing due to anatomic changes during treatment or unexpected delays in obtaining diagnostic MR appointments.

**Conclusions:**

In four pediatric patients treated with MRgRT, treatment was well tolerated with no severe acute effects. At our center, most pediatric patients are treated with proton therapy, but the cases selected for MRgRT demonstrated significant organ at risk sparing compared to alternative modalities. In particular, MRgRT may provide advantages for thoracic/abdominal/pelvic targets using gated delivery and adaptive replanning, but selected patients treated with fractionated radiotherapy may also benefit MRgRT through superior organ at risk sparing.

## Introduction

Magnetic resonance-guided radiotherapy (MRgRT) provides enhanced soft tissue visualization compared to computed tomography (CT) and the potential for an online adaptive workflow, which may enable safer dose escalation for tumors adjacent to dose-limiting organs at risk (OAR) without increasing toxicity. Additional benefits include improvements in daily setup accuracy, the ability to reduce planning target volume (PTV) margins for some disease sites, use of continuous cine tumor motion tracking and beam gating, and application of respiratory breath-hold techniques to abrogate tumor motion. In adults, MRgRT has been applied in stereotactic ablative radiotherapy (SABR) for inoperable pancreatic carcinoma and oligometastatic lesions in the abdomen, pelvis, liver, and adrenal glands with favorable early outcomes (1, 2).

In children (defined as < 21 years of age in this study), MRgRT may provide similar opportunities to improve the therapeutic ratio with a potentially greater emphasis on reducing late adverse effects of radiotherapy. In 2020, a survey of twelve current and future users of MRgRT systems in International Society of Paediatric Oncology (SIOP) and Children’s Oncology Group (COG) radiotherapy centers examined the potential benefits of MRgRT in pediatric patients (3). While the survey identified several clinical scenarios and tumor sites where MRgRT was expected to improve clinical outcomes and toxicities, experience with MRgRT in children remains limited. To date, the published experience for the use of MRgRT in pediatric patients is limited to two case reports (4, 5), which is likely influenced by the low number of MRgRT facilities and prioritization of other modalities, such as proton therapy (PT), in this population. The purpose of this investigation is to report on the use of MRgRT for treatment and imaging purposes at our institution during a four-year period, discuss potential applications for this technology at a large center with varied radiotherapy modalities, and describe lessons learned from treating pediatric patients with MRgRT.

## Materials and methods

After obtaining institutional review board (IRB) approval, we retrospectively reviewed all patients treated with MRgRT on the MRIdian (ViewRay, Oakwood Village, OH) linear accelerator (MR Linac) at a single institution between 4/2018 and 4/2022. We also reviewed all pediatric and young adult patients (< 21 years of age) treated at our institution during the same time interval and identified all who received either imaging or treatment using the MR Linac.

All patients underwent simulation and treatment in the supine position. Every simulation comprised both a 0.35 T balanced steady-state free precession sequence (TrueFISP) MR scan acquired over 17-25 seconds on the MR Linac followed by a CT simulation. Patients treated with conventional fractionation using a non-adaptive workflow were simulated arms up or arms down based on the disease site and at the discretion of the radiation oncologist. For abdominal and thoracic tumors undergoing SABR, simulation was performed either with both arms down or one arm raised above the head for comfort and reproducibility; this was important particularly for patients undergoing daily online adaptive replanning and treatment in breath hold. For SABR, our treatment planning and delivery approach were previously described (6). Fiducial markers and oral/intravenous contrast were not used given that gross disease and OARs were well visualized during simulation and treatment. Target volume and OAR delineation and treatment planning were performed on the MR simulation scan. When appropriate, and based on the disease site treated, a clinical target volume (CTV) was added surrounding the gross tumor volume (GTV) at the discretion of the radiation oncologist. The PTV margin consisted of an isotropic 3 mm expansion of GTV or CTV (if present).

Prior to each daily treatment, GTV was used to define the tracking region of interest in the sagittal plane. Continuous cine imaging and real-time tumor tracking were applied, and treatment was automatically held if > 3-5% of the tracking region of interest was displaced by > 3 mm from its original location (e.g. outside of the tracking boundary). In SABR cases, mid-inspiration breath hold was preferred over deep inspiration breath hold respiratory gating and free breathing to improve treatment efficiency and decrease the time the patient was required to be in the MR Linac. On-table adaptive replanning was performed in SABR cases where OAR anatomy was expected to change from day to day and dose constraints would be exceeded. The target volumes and critical OARs within 2 cm of the PTV were recontoured every day and replanning was performed if deemed medically necessary based on predicted dose from the initial plan recalculated on the anatomy of the day. The highest priority for all delivered treatments was to ensure that OAR constraints were met, even if target coverage was compromised. During planning, treatment plans were optimized to deliver 95% of the prescription dose to 100% of the PTV. In the event that organ at risk constraints could not be met with this dose coverage, OAR constraint priorities were met and undercoverage was accepted. During daily online adaptive replanning, we employed an isotoxicity planning approach, where treatment plans were normalized to the nearest OAR dose constraint, typically for the nearest gastrointestinal (GI) OAR. Pretreatment patient-specific quality assurance was performed before delivery of the first planned fraction in all cases and was performed prior to each daily fraction in all plans that underwent online adaptive replanning.

Clinical and radiographic data from baseline and routine follow-up, including patient and tumor characteristics, treatment details, acute and chronic toxicities, and disease response, were collected (by MDH, RH, and KVW) and entered into a coded electronic database. Electronic medical records were also reviewed from the primary pediatric oncology teams for assessment of toxicities. Patients were seen 3 months after MRgRT and then every 3-4 months for routine care in our clinic. Treatment response was evaluated with Response Evaluation Criteria in Solid Tumors (RECIST) version 1.1 criteria. Early and late toxicities were prospectively recorded weekly during MRgRT and then at each radiation oncology follow-up visit using Common Terminology Criteria for Adverse Events (CTCAE) version 5. Acute toxicity was defined as any toxicity occurring during or within 90 days after completing MRgRT.

Descriptive statistics were used to illustrate patient allocation between various treatment modalities in our department and patient-specific outcomes. Local control (LC) was defined as the absence of in-field treatment failure. Overall survival (OS) was determined by the time to death from any cause with censorship at the date of last follow-up. The data presented here comprise all follow-up data up to the close-out date of September 5, 2022.

## Results

Between 4/2018 and 4/2022, 535 patients received MRgRT in at our center. Of this total, 405 patients (75.7%) received SABR, defined as doses ≥ 6 Gy delivered in ≤ 10 fractions. Within the SABR cohort, 370 patients were treated with ablative dosing using 5 or fewer fractions while 35 received 6-8 fractions. The two reasons patients did not receive ≤ 5 fractions were if insurance did not approve five-fraction SABR (in this event, patients most often received 40-50 Gy in 6 fractions) or if the radiation oncologist selected a more gently fractionated ablative regimen, such as 60 Gy in 8 fractions for central lung tumors. The most common sites treated in this cohort using SABR were inoperable pancreatic cancer (26.5%), lymph node metastases (16.1%), hepatobiliary tumors (10.3%), and adrenal metastases (9.0%). In this dataset, 69 MRgRT patients (12.9%) received conventional fractionation, most commonly for lung and GI tumor sites treated with definitive intent.

During this four-year period, 347 distinct courses of external beam radiotherapy were delivered to pediatric and young adult patients who were < 21 years of age. This included 28 patients treated with cranial stereotactic radiosurgery (8.1%) and 29 who received total body irradiation as part of the conditioning regimen for hematopoietic cell transplantation. As a result, a total of 290 courses of fractionated external beam radiotherapy were delivered during this interval, including 217 (74.8%) with proton therapy.

In this same period, four pediatric patients received MRgRT. One patient with metastatic Ewing sarcoma received SABR for a lung metastasis in the left lower lobe abutting the diaphragm. For this patient daily adaptive replanning was adopted to meet OAR constraints to the adjacent stomach. For this patient daily adaptive replanning was adopted to meet OAR constraints to the adjacent stomach. A second patient received non-adaptive SABR for a single liver metastasis with gated beam delivery. Two patients received MRgRT to 30 Gy in 10 fractions for metastatic ALK-rearranged non-small cell lung cancer and neuroblastoma. Fourteen other patients had imaging alone performed on the MR Linac for either image fusion or quality assurance during treatment. Twelve underwent MR imaging without contrast for brain tumors to assess for tumor/cystic changes during treatment. Two additional patients who were subsequently treated using other radiotherapy modalities underwent MR simulation for target volume delineation and organ at risk sparing due to observed anatomic changes or unexpected delays in obtaining diagnostic MR appointments.

Below, we describe four clinical cases from this cohort that illustrate the potential clinical applications for MRgRT in pediatric cancer patients. In addition, we illustrate one example patient in which the MR Linac was used for image fusion and offline adaptive replanning in a patient treated with proton therapy.

## Clinical cases and outcomes

### Case 1. Lung metastasis near the diaphragm

The patient is an 18-year-old female with recurrent Stage IV Ewing sarcoma with three oligoprogressive lung metastases. Prior therapy included systemic chemotherapy with vincristine, doxorubicin and cyclophosphamide (VDC) alternating with ifosfamide and etoposide (IE) according to Children’s Oncology Group (COG) study AEWS1031. She also received surgery and postoperative radiotherapy for a primary tumor in the sacrum and comprehensive metastatic site radiotherapy, including whole lung irradiation to 15 Gy in 10 fractions. The patient relapsed 18 months following completion of primary treatment with metastatic disease in the lungs and recurrence of the primary tumor in the Lumbar spine. She received vincristine, irinotecan, and temozolomide (VIT) chemotherapy with partial response. After chemotherapy, three residual lung metastases remained. Due to prior whole lung irradiation, stereotactic ablative radiotherapy was recommended to 35 Gy in 5 fractions (7, 8).

**Figure 1:**
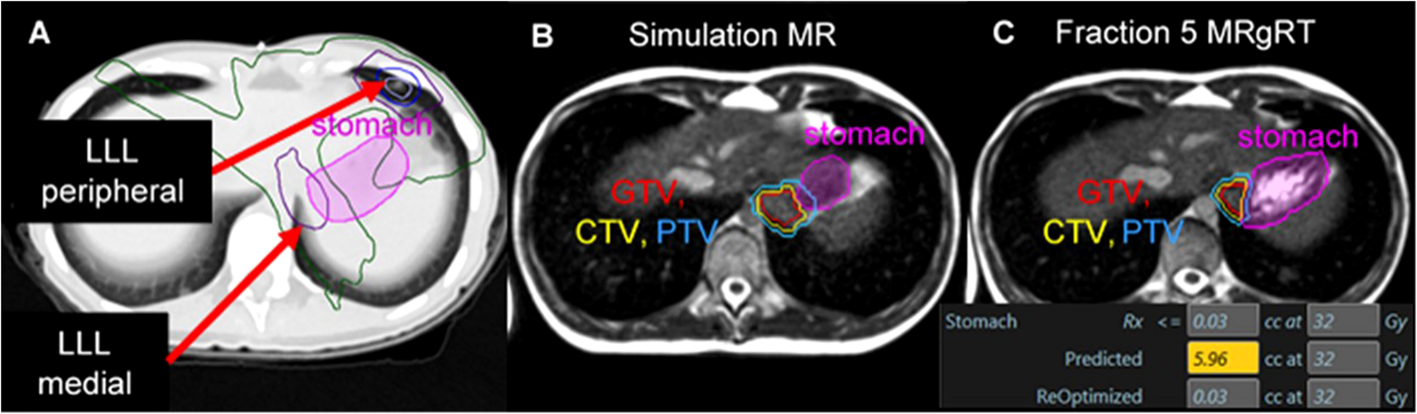
A patient with metastatic Ewing sarcoma with two left lower lobe lesions where two SABR plans resulted in dose overlap within the stomach. Isodose lines: Yellow = 40 Gy, Blue = 33 Gy, Green = 20 Gy, Purple = 10 Gy all over 5 fractions **(A)**. A significant change in stomach anatomy was observed during daily cine imaging between simulation **(B)** and each treatment fraction **(C)**. Online adaptive replanning was performed to enable the plan of the day to meet dose constraints for the stomach; in this example, the predicted dose on the anatomy of the day would have exposed 5.96 cc of the stomach to ≥ 32 Gy, while the re-optimized plan improved GTV/PTV coverage and met dose constraints.

The patient underwent four-dimensional CT simulation for radiation treatment planning. Two metastases were peripherally located, including one in the anterior left lower lobe (LLL) as illustrated in **Figure 1**. On CT simulation, tumor excursion was < 8 mm for these lesions. Based on the scoring system proposed by Seravalli and colleagues for the use of MRgRT in pediatric patients, these two lesions were assessed to have a modest potential benefit with MRgRT (3). Based on modest benefit in terms of reduction of total lung dose, these two lesions were treated with SABR using volumetric-modulated arc therapy (VMAT) stereotactic delivery.

In comparison, the third lung metastasis was in the posterior LLL and was near the heart, esophagus, and stomach. This lesion had a maximal tumor excursion of 15 mm during breathing with abdominal compression. Based on proximity to radiosensitive OARs and tumor motion > 10 mm, a strong benefit from MRgRT was predicted. MR simulation was performed with the left (ipsilateral) arm above the head to enable left-sided beams during treatment; the right arm was positioned at the patient’s side. This third metastatic lesion was treated with MRgRT in mid-inspiration breath hold with daily online adaptive replanning. OAR dose objectives were set to keep the stomach D_0.03cc_ < 32 Gy. This constraint included the dose contribution from SABR to the anterior LLL delivered using VMAT. **Figure 1** illustrates the large magnitude interfraction change observed in the stomach between simulation and the 5^th^ treatment fraction and the resulting dose constraint violation to the stomach based on the anatomy of the day. Online adaptive replanning was performed for all 5 fractions due to predicted dose constraint violations to the stomach.

No adverse toxicities were observed during or after SABR to these metastatic lesions through 11 months of follow-up. The patient had a radiographic complete response of all three treated lesions on last imaging 10 months after SABR. The patient developed further disease progression at the site of the primary tumor and was treated with chemotherapy and palliative reirradiation 3 months after SABR. At last follow-up, she had active disease at other non-lung sites and continued palliative chemotherapy.

### Case 2. Liver metastasis

The patient is a 7-year-old female with a history of rhabdomyosarcoma who presented with a solitary site of metastatic disease in the caudate lobe of the liver. The patient was diagnosed at age 5 with a primary tumor in the distal lower extremity with biopsy-proven popliteal nodal involvement. The patient received systemic chemotherapy according to COG ARST0431 with vincristine and irinotecan (VI), followed by VDC alternating with IE, and then vincristine, dactinomycin, and cyclophosphamide (VAC) alternating with VI. Local therapy included surgery for the primary tumor in the distal calf followed by adjuvant radiotherapy due to nodal involvement. At relapse, she presented with a solitary site of metastatic disease in segment IV of the liver. She received VIT chemotherapy with partial response and no new evidence of metastatic disease. She was referred for consideration of consolidative radiotherapy.

**Figure 2:**
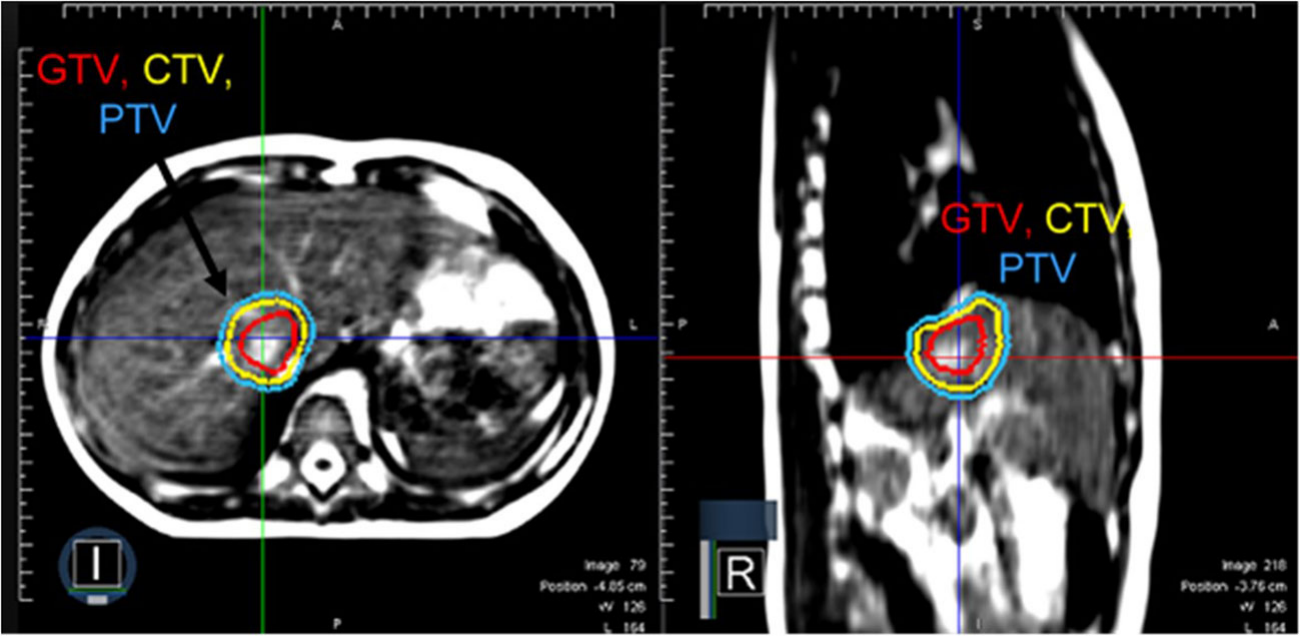
A patient with metastatic rhabdomyosarcoma with a solitary liver metastasis who received SABR using MRgRT. Continuous cine imaging and gated delivery during mid-inspiration breath hold enabled treatment with PTV expansion of 3 mm. No ITV was used.

Based on the tumor location and the anticipated tumor excursion during breathing, SABR using MRgRT was recommended. During MR simulation, both arms were placed at the patient’s side for comfort and treatment compliance. On CT simulation, the estimated tumor excursion during breathing was between 15 to 20 mm. The patient was treated with SABR to 40 Gy in 5 fractions using MRgRT for margin reduction and improved soft tissue visualization. **Figure 2** illustrates that the 0.35 T MRI clearly distinguished tumor from the normal liver without contrast. The patient was planned for treatment with gated delivery in mid-inspiration breath hold using continuous cine MR imaging for tumor tracking in the sagittal plane at 4 frames per second. No internal target volume (ITV) expansion was added. During treatment, the patient proved largely unable to adhere to mid-inspiration breath hold as instructed by the radiation therapists. The patient was coached during treatment with suboptimal compliance. Treatment was still delivered on MRgRT with gated beam delivery when the tumor was in position. The patient tolerated MRgRT with no adverse effects during treatment apart from poor compliance.

At three months, the treated lesion in the caudate lobe of the liver demonstrated a complete radiographic response on imaging. However, 6 additional lesions were identified in the liver in addition to disease in the pancreatic head. Additional salvage therapies, included pazopanib and nivolumab, were given. The patient died with disease 7 months after completion of SABR. No adverse events were observed or reported during this follow up interval. Final CT-based imaging performed within one week of the patient’s death demonstrated no clear evidence of disease recurrence in the caudate lobe of the liver.

### Case 3. Metastatic neuroblastoma of the mandible

The patient is a 7-year-old male with high-risk neuroblastoma with a painful metastasis involving the right mandible. He was initially diagnosed with Stage IV disease and received high-risk chemotherapy according to COG ANBL0532. He received consolidative proton therapy to 21.6 Gy RBE in 12 fractions to the abdomen and a metastasis in the left temporal bone of the skull that remained positive on functional imaging before high dose chemotherapy with stem cell rescue. At first recurrence, he developed metastatic disease in multiple bones and was treated with salvage chemotherapy and dinutuximab. He was referred for radiotherapy for a painful mass involving the mandible. Based on limited volume disease, palliative radiotherapy to 30 Gy in 10 fractions was recommended. Based on the scoring system by Seravalli and colleagues, we estimated that MRgRT would provide limited potential benefit in terms of tumor control for this dose regimen but anticipated a modest benefit may be derived from sparing the oral cavity compared to conventional linear accelerator (3).

**Figure 3:**
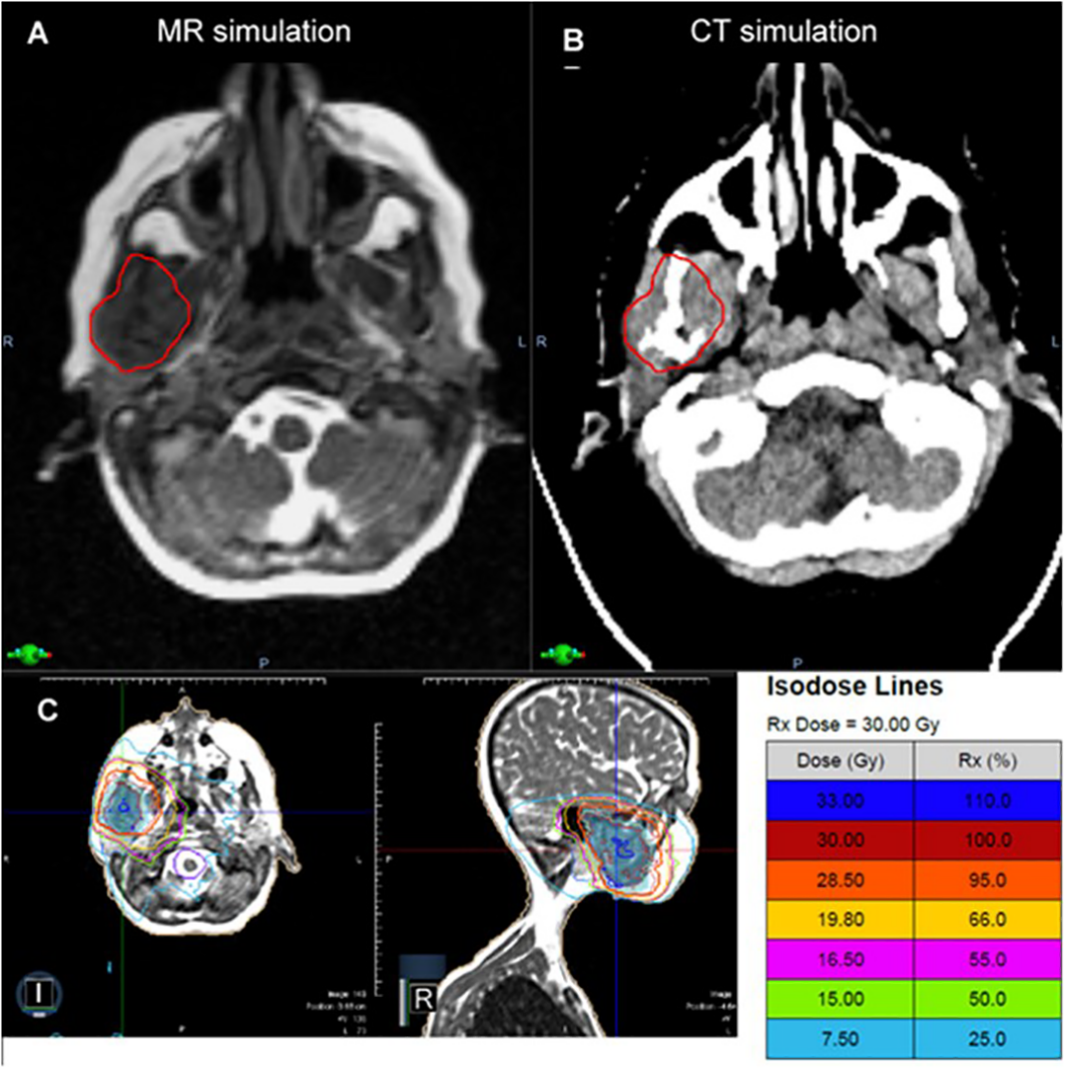
In a patient with metastatic neuroblastoma involving the mandible, the MR simulation **(A)** provided superior visualization of the tumor relative to the adjacent masseter and pterygoid muscles compared to CT **(B)**. The patient was treated with a 3D conformal MRgRT plan **(C)**, which provided favorable sparing of the oral cavity and the ipsilateral and contralateral salivary glands with similar plan quality to IMRT.

The patient underwent MR and CT simulation with a thermoplastic mask and moldcare pillow. **Figure 3** demonstrates that the MR Linac significantly improved soft tissue visualization and permitted differentiation between the tumor and the adjacent masseter and pterygoid muscles. As a result, MRgRT enabled more precise target volume delineation than would be feasible with CT-based planning, where GTV would have been overestimated. The patient was treated with a 3D conformal MRgRT plan using 9 fields that delivered a mean dose of 18.5 and 17.5 Gy to the ipsilateral parotid and submandibular glands, 9.0 Gy to the oral cavity, and < 5 Gy to the contralateral parotid and submandibular glands. The MRgRT plan enabled significant reduction in OAR dosing, particularly to the oral cavity, compared to 3D conformal plan on a conventional linear accelerator. Due to the treatment planning process for MRgRT, the MR Linac 3D conformal plan was similar in quality to an IMRT plan without excess cost to the healthcare system. At follow up visits at 3 and 6 months, the patient denied xerostomia, dysgeusia, oral mucositis, and pain, which are commonly experienced by patients following palliative radiotherapy to this region.

### Case 4. Metastatic non-small cell lung adenocarcinoma in a lifelong non-smoker

**Figure 4:**
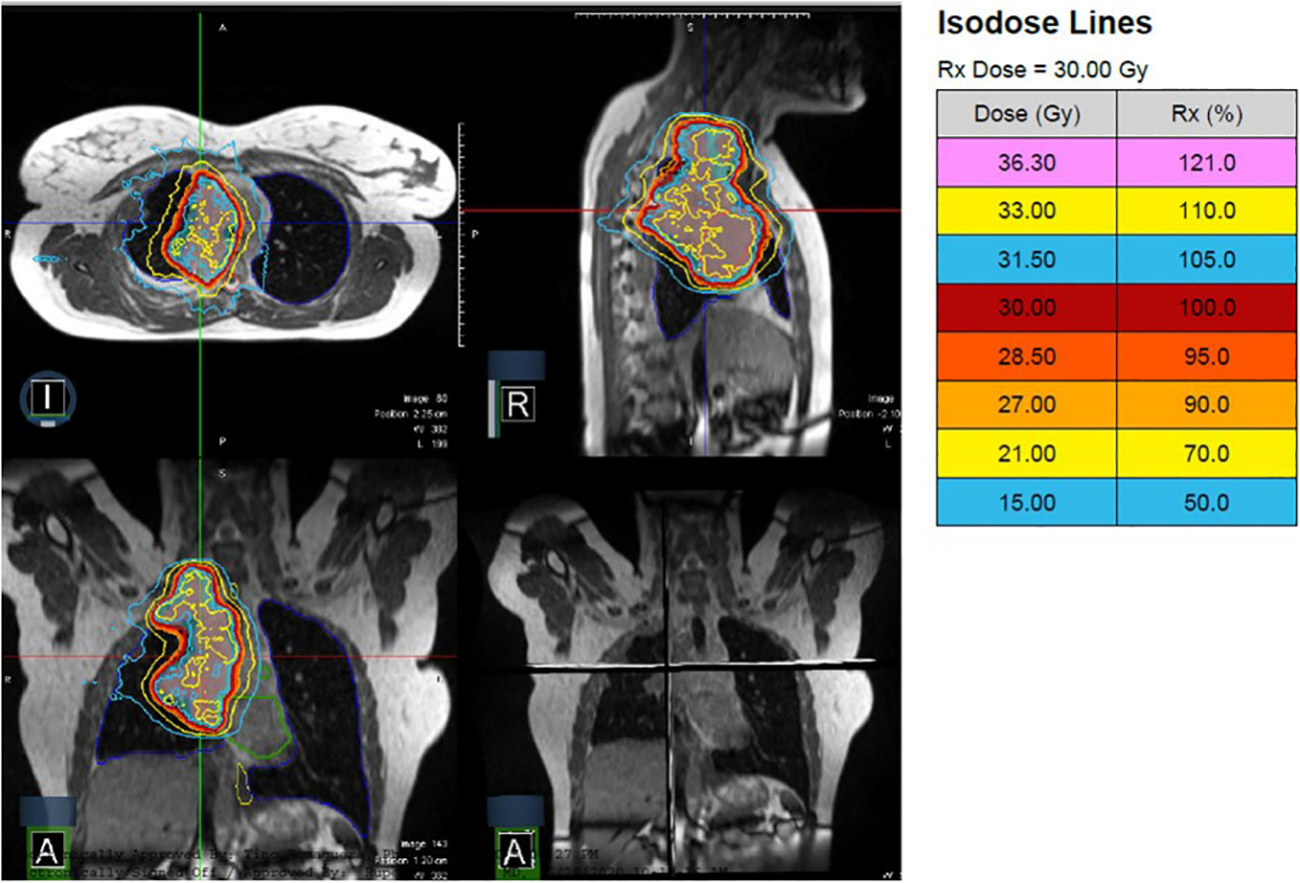
A patient with Stage IV ALK-rearranged non-small cell lung cancer and a 10 cm primary tumor received 30 Gy in 10 fractions with MRgRT. Continuous cine imaging and gated delivery during mid-inspiration breath hold enabled treatment with PTV expansion of 3mm. No ITV was used.

The patient is a 19-year-old non-smoker with Stage IV ALK-rearranged non-small cell lung cancer, who presented with right neck and chest pain and Horner’s syndrome. Imaging demonstrated a 10 cm soft tissue mass abutting the right mediastinum and displacing the right heart border, multiple pleural-based soft tissue masses, enlarged mediastinal and right supraclavicular nodes, and bone metastases in C5, C7, T1, T3, and T4 with involvement of the neural foramina at T3-T4. Due to pain, she was referred for radiotherapy and received 30 Gy in 10 fractions with MRgRT using step-and-shoot IMRT. **Figure 4** depicts the MRgRT plan, which delivered a mean dose of 7.77 Gy to the heart, 7.92 Gy to the lungs, and 16.78 Gy to the esophagus. The volume of esophagus receiving prescription dose was 18%.

Following treatment, the patient developed Grade 1 esophagitis, managed with dietary changes and increased fluid intake. No pain medications were needed. She initiated alectinib for systemic therapy. At two months following radiotherapy, a partial response was documented on imaging with a 50% volumetric reduction in the dominant mass. Last clinic and imaging follow up was 2 years and 3 months following MRgRT, when the patient had no clear evidence of residual tumors in the lung and sclerotic bone lesions, consistent with treated tumor.

### Weekly imaging on the MR Linac and offline adaptive replanning in a craniopharyngioma patient receiving proton therapy

**Figure 5:**
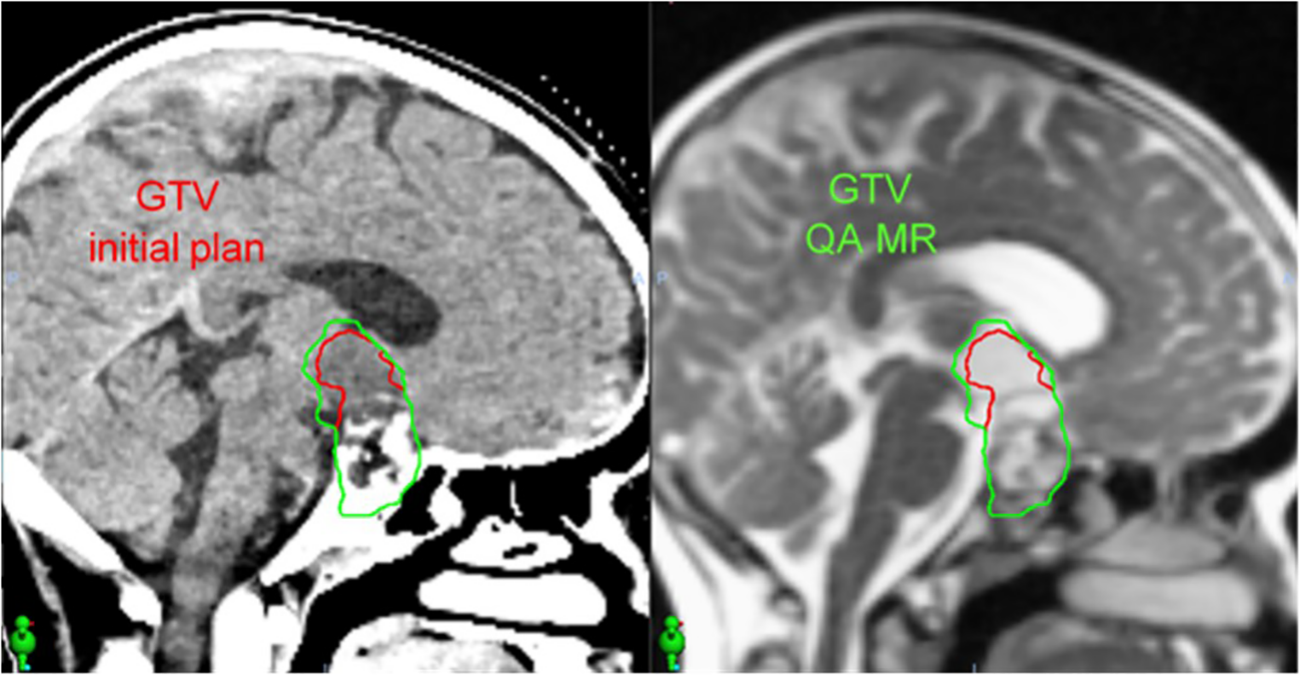
In a patient with craniopharyngioma, an on-treatment MR acquired using the MR Linac (right) 22 days after CT simulation (left) demonstrated increased size of the tumor/cyst. This MR image set was used to perform offline adaptive replanning.

The patient is a 10-year-old male with craniopharyngioma who received intensity-modulated proton therapy to 54 Gy RBE in 30 fractions. Proton therapy was recommended based on the favorable prognosis and significant reduction in total integral dose and hippocampus and temporal lobe sparing. Weekly on-treatment MR imaging was obtained on the MR Linac to evaluate changes in the tumor/cyst and the need for offline adaptive replanning. **Figure 5** demonstrates the CT simulation and the MR acquired on the MR Linac during week 1 of proton therapy (22 days following simulation), which demonstrated that the tumor/cyst had increased in size and now abutted the original CTV contour. Offline adaptive replanning was performed. The patient received two additional fractions using the initial proton therapy plan and then began the new plan three days after MR imaging.

In our center, weekly MR is feasible on the MR Linac either before or after proton therapy and eliminates the need for a separate appointment in radiology for a diagnostic MRI without contrast. This is a more efficient use of patient time and cancer center resources, given that the weekly appointments on the MR Linac last approximately 10 minutes and are more easily coordinated with proton therapy treatment times. This provides significant time savings for the patient and family compared to an appointment in radiology and still permits excellent tumor differentiation for adaptive replanning (9).

## Discussion

MRgRT is a relatively new modality in radiation oncology, which is growing in utilization as more systems are brought online. Early results suggest that MRgRT may lead to clinical benefits in selected adults across several disease sites, including inoperable pancreatic cancer treated with SABR with survival rates that compared favorably to historical controls (1) and prostate SABR with improved toxicity rates compared to cone-beam CT-based delivery (10). The current experience for pediatric cancers, however, remains particularly limited (4, 5). To our knowledge, this is the largest published experience of MRgRT in pediatric and young adult patients, amounting to only four patients during the first four years of utilization at our institution.

The MOMENTUM academic industrial collaborative group recently reported early outcomes of MRgRT patients treated on a prospective registry between February, 2019 and October, 2020. In this first report, 943 adult patients (age 21-93) were treated at 7 institutions in Europe, Canada, and the United States, and 415 (44.0%) had acute toxicity data at 3-month follow-up. The observed rate of grade 3 toxicity was 4% in patients treated for a wide range of indications including prostate cancer, oligometastatic lymph nodes, brain tumors, and rectal cancer; the majority of patients received ≤ 5 fractions (11). While clinical experience with MRgRT is growing in adult tumors, greater effort and planning is needed to bridge the gap on clinical development in pediatrics.

Due to the rarity and diversity of pediatric tumors, multi-institutional collaboration by all centers treating pediatric patients with MRgRT is a significant unmet need. To this end, a pediatric MRgRT working group of twelve members across SIOPE and COG-affiliated radiotherapy departments, including three ViewRay MRIdian and nine Elekta Unity (Elekta, Crawley, UK) users, was established in June, 2021 (3). Given the small number of pediatric patients who will receive MRgRT at each center, enrolling all patients into a registry and prospectively tracking outcomes will be important to build clinical expertise.

While the experience presented here only adds five scenarios where the MR Linac was applied in clinic, we observed three important findings. First, MRgRT can be applied in varied clinical scenarios, but the same advantages identified in adult patients also exist in children, including improved soft tissue visualization, gated delivery plus real-time tumor tracking, and online adaptive replanning to meet critical OAR constraints. Cases 1 and 2 in this report describe tumors treated with SABR that were characterized by significant motion and proximity to luminal GI OARs where gated delivery and online adaptive replanning enabled superior target coverage without an ITV. Second, patient selection must be carefully considered both for MRgRT utilization and based on the relative benefits of other available modalities in the department. At our center, SABR, most commonly with online adaptive replanning, comprises approximately 75% of MRgRT cases. Given that each treatment fraction generally lasts 50-60 minutes with online adaptive replanning, limited slots remain for treatment of patients with more modest benefits from MRgRT. This is also illustrated in Case 1 of this report. While the metastatic lesion near the stomach received MR-guided SABR, two other lesions with more limited respiratory excursion and without the need for online adaptive replanning were treated with CBCT-based SABR. Further, most pediatric patients at our center receive proton therapy for curative intent tumors in order to reduce late effects following treatment. If proton therapy were not accessible to patients at our center, the utilization of MRgRT may have been different than observed here, but the authors find that MRgRT is unlikely to replace proton therapy in the vast majority of cases at our institution. At our institution, patient selection is largely driven by the treating physician. Cases 3 and 4 present two patients who could have received equally efficacious treatment without MRgRT but may have potentially benefited from OAR sparing and resulting reduction in acute toxicities with this approach.

Many challenges with MRgRT utilization are similar in adults and children. One potential barrier is longer treatment times, particularly when using an online adaptive workflow. Patients should be selected who will be able to hold still for the requisite amount of time to complete treatment. Careful patient positioning and immobilization at simulation should also be enacted to improve patient comfort and compliance. Future improvements, including automatic contouring and planning capabilities may reduce treatment times and minimize this challenge with MRgRT (12). Similarly, technological improvements in MRgRT such as volumetric arc delivery may also reduce delivery times and improve plan quality (13). Real-time respiratory gating is currently available only in a subset of MRgRT units, which may reduce plan quality and the ability to deliver ablative dosing for some tumor locations. In the future, the integration of respiratory gating capabilities in all MRgRT systems may enable greater utilization in abdominal and pelvic tumors near GI OARs. Patient selection is critical to identify patients who will gain the most from MRgRT. Clinicians should consider the importance of (1) soft tissue visualization, (2) respiratory motion management, and (3) proximity to OARs and the ability to spare them using MRgRT with or without online adaptive replanning compared to other radiotherapy modalities.

**Figure 6:**
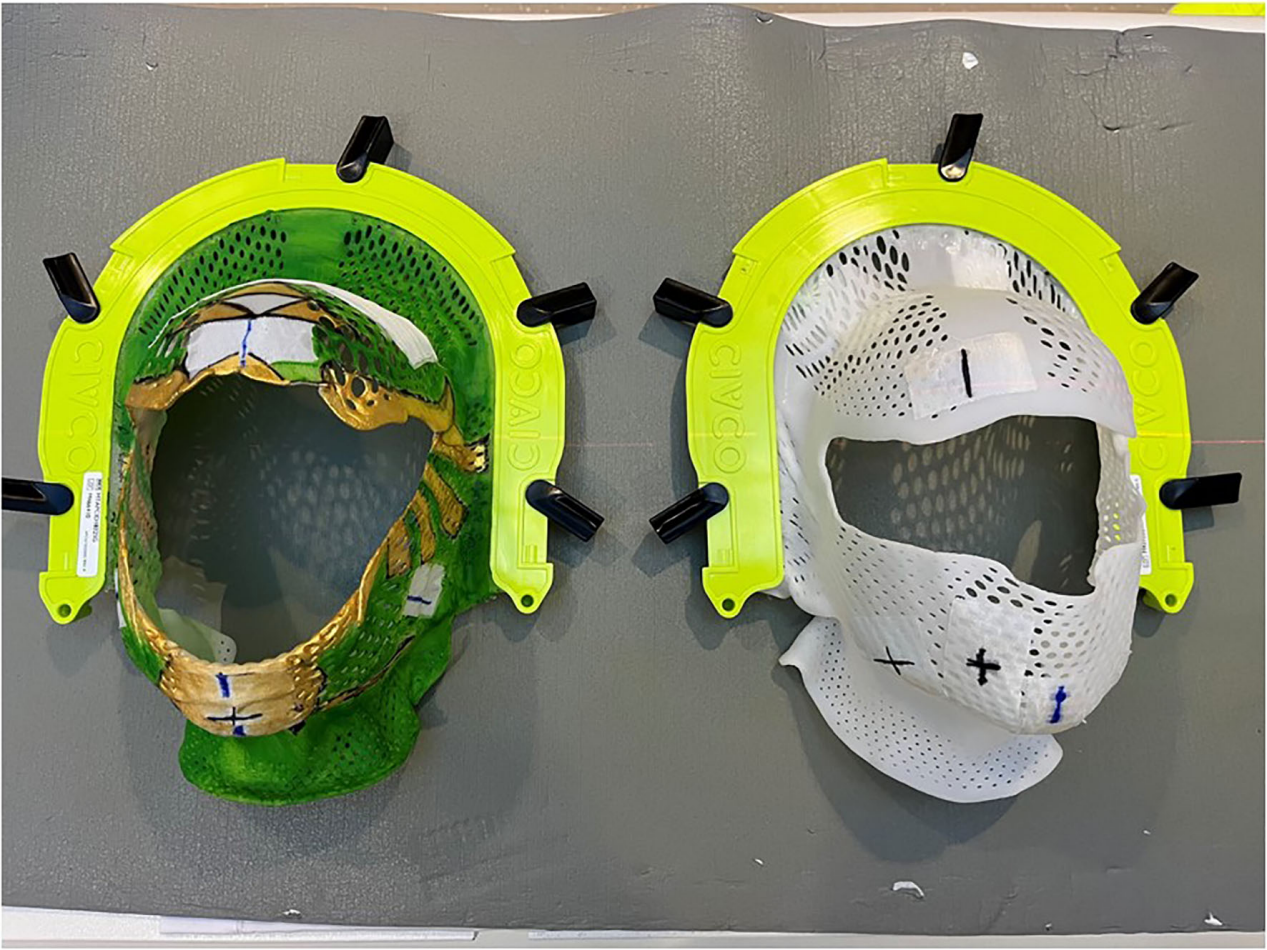
This image illustrates a mask used to treat a pediatric patient for metastatic neuroblastoma that has been decorated with paint that contained metallic components. All devices used during MRgRT must be MR compatible. In this case, the patient was re-simulated before treatment with an unpainted mask.

Other barriers to the use of MRgRT in pediatric patients are more exclusive to children. For example, pediatric patients more commonly use anesthesia during radiotherapy than adults. Potential users should consider anesthesia needs if planning to treat such patients with MRgRT, including the use of MR-compatible anesthesia and patient monitoring equipment within the vault. Supportive care resources, such as an audiovisual entertainment system and a certified child life specialist can also assist with treatment compliance and emotional adjustment for patients who will undergo MRgRT (14, 15). Finally, staff should ensure that all equipment and devices used are compatible with MRgRT. For example, the patient treated in Case 3 had to be re-simulated prior to treatment because metallic paint was applied to the thermoplastic mask to create a superhero image at the request of the patient. **Figure 6** illustrates both the decorated mask and the new one that was ultimately used for treatment. Metallic paint can induce heating in the MR Linac vault and could potentially result in skin burns. This case example provides and important opportunity to stress the importance of MR safety education and awareness in developing a MRgRT program.

We acknowledge several limitations in this published work. First, due to its retrospective design and the patient population treated, the duration of follow-up was generally short and was often limited due to death from disease. Retrospective studies can potentially underreport treatment-related toxicities. We strived to mitigate this limitation by prospectively evaluating toxicities at each patient visit during and after treatment as a matter of routine care. In addition, our study period included several years during the COVID-19 pandemic. While this reality certainly altered healthcare delivery, patient follow-up arguably was not adversely impacted by the pandemic, due in large part to the incorporation of telehealth conferences with patients during follow-up. Distance from our facility and appointments with other physicians represent common barriers for patients to attend follow-up clinic in person. In contrast to disruption, the typically short courses delivered on MRgRT may have been more practical for patients compared to longer courses of radiation that may have been given without MR Linac and follow-up was maintained or may have been arguably improved with patients using telehealth services to make some of their appointments in our clinic. Second, patient-reported outcomes are important measures of treatment tolerance and toxicity and were not collected in this analysis. The future prospective pediatric MRgRT registry should consider including such metrics to better understand patient quality-of-life following MRgRT. Finally, the small incidence of pediatric tumors overall and the scarcity of pediatric patients who will receive MRgRT will lead to limited patient numbers and considerable heterogeneity in clinical outcomes. This underscores the importance of prospective registries to build clinical knowledge and technical skill in the application of MRgRT in new patient populations, including children.

In summary, we report four clinical cases treated with MRgRT at a single institution and an example patient where MR Linac was used for mid-treatment imaging and offline adaptive replanning during treatment. The four patients treated with MRgRT tolerated treatment well and without any Grade 2 or higher toxicities following treatment. Our manuscript adds to the body of literature on the use of MRgRT in pediatric patients, illustrates several clinical scenarios where MRgRT may be used, and describes several lessons learned that are pertinent to future users of this novel radiotherapy treatment strategy.

## Data availability statement

The raw data supporting the conclusions of this article will be made available by the authors, without undue reservation.

## Ethics statement

The studies involving human participants were reviewed and approved by Miami Cancer Institute IRB. Written informed consent from the participants’ legal guardian/next of kin was not required to participate in this study in accordance with the national legislation and the institutional requirements.

## Author contributions

The authors affirm their contributions to the paper as follows: Study conceptualization: MH, KM, and MC. Data curation: MH, RH, KM, and KV. Data analysis: MH and KM. Writing – original draft: MH, KV, KM, and MC, Manuscript editing and review: All authors. All authors contributed to the article and approved the submitted version.
